# Handgrip Maximal Voluntary Isometric Contraction Does Not Correlate with Thenar Motor Unit Number Estimation

**DOI:** 10.1155/2012/187947

**Published:** 2012-05-09

**Authors:** Arun Aggarwal

**Affiliations:** Department of Neurology, Concord Hospital, Concord, NSW 2139, Australia

## Abstract

In slowly progressive conditions, such as motor neurone disease (MND), 50–80% of motor units may be lost before weakness becomes clinically apparent. Despite this, maximal voluntary isometric contraction (MVIC) has been reported as a clinically useful, reliable, and reproducible measure for monitoring disease progression in MND. We performed a study on a group of asymptomatic subjects that showed a lack of correlation between isometric grip strength and thenar MUNE. Motor unit number estimation (MUNE) estimates the number of functioning lower motor neurones innervating a muscle or a group of muscles. We used the statistical electrophysiological technique of MUNE to estimate the number of motor units in thenar group of muscles in 69 subjects: 19 asymptomatic Cu, Zn superoxide dismutase 1 (SOD 1) mutation carriers, 34 family controls, and 16 population controls. The Jamar hand dynamometer was used to measure isometric grip strength. This study suggests that MUNE is more sensitive for monitoring disease progression than maximal voluntary isometric contraction (MVIC), as MUNE correlates with the number of functional motor neurones. This supports the observation that patients with substantial chronic denervation can maintain normal muscle twitch tension until 50–80% of motor units are lost and weakness is detectable.

## 1. Introduction

Motor neurone disease (MND) is considered to be a group of generally fatal, progressive neurodegenerative disorders. The disease is characterised pathologically by progressive degeneration and loss of motor neurones in the anterior horn cells of the spinal cord, motor nuclei of the brainstem, and the descending pathways within the corticospinal tracts [1]. It is primarily a condition of middle to late life, with onset of symptoms between the ages of 50 and 70. The overall median survival is 4.0 years from the onset of symptoms [2]. When disorders are recent or rapidly progressive, motor neurone loss results in weakness and wasting. In slowly progressive denervating diseases such as MND, loss of more than 50–80% of motor units may occur before weakness becomes clinically apparent [3].

In MND, needle electromyography may reveal changes of chronic reinnervation, but provides little direct evidence to the extent of motor neurone and axonal loss. The supramaximal compound muscle action potential (CMAP) amplitude also provides little direct evidence of the extent of motor neurone loss as a normal CMAP amplitude might mistakenly be thought to indicate that motor neurone loss has not occurred [4].

Motor unit number estimation (MUNE) has been shown to be a more reliable method for following changes in neurogenic disorders than the CMAP amplitude [5]. It estimates the number of functioning lower motor neurones innervating a muscle or a group of muscles. It is an indirect measure of motor neurone loss, rather than a measure of pathology.

The concept of MUNE originated in 1967 by McComas et al. [6]. The principle of MUNE is that the mean single motor unit amplitude (SMUP) can be measured; it is possible to obtain an estimate of the total number of motor units in the muscle [7]. The results were comparable with estimates of alpha motor fibres obtained by counting axons in specimens of motor nerves [8].

MUNE has been performed in a number of different ways, each with their advantages and limitations. Each method measures both the average SMUP and the size of the CMAP amplitude obtained with maximal stimulation of a motor nerve. The statistical MUNE technique was used in this study to determine thenar MUNE [9].

There are a number of methods that have been developed to quantify maximal voluntary isometric contraction (MVIC) more objectively, as the traditional neurological examination is inadequate for documenting motor performance reliably [10, 11]. These include an electronic strain-gauge tensiometer and a hand-held Jamar hydraulic dynamometer [12]. These have been proposed as a quantitative measure for monitoring disease progression in MND [10].

We used the Jamar hand dynamometer to measure isometric grip strength to determine whether this correlated with the number of functional motor neurones in the thenar group of muscles as measured by the statistical MUNE method in a group of asymptomatic subjects, all of which had MRC grade 5/5 power on clinical neurological examination.

## 2. Methods

Family members of known SOD-1-positive families were contracted and recruited into our original study [13]. 69 subjects agreed to participate. 19 were asymptomatic SOD 1 mutation carriers, 34 age and sex matched SOD-1-negative family controls and 16 normal population controls. The study was approved by the CSAHS Ethics Review Committee.

Numerous studies have shown that MUNE can be used to monitor change over time in MND patients when used by an experienced technician, despite evaluator bias [14]. We used the statistical MUNE technique for this study [9]. This technique has undergone a number of revisions since its original description to take into account motor unit instability [15, 16].

The motor unit number estimate is calculated by:

(1) $$\;\text{MUNE}=\frac{\text{Maximum}\;\text{CMAP}\;\text{amplitude}\;\left(\text{or}\;\text{area}\right)}{\text{Average}\;\text{single}\;\text{motor}\;\text{unit}\;\text{potential}\;\left(\text{SMUP}\right)\;\text{amplitude}\;\text{or}\;\text{area}}.$$

In this study, we estimated motor unit numbers from abductor pollicis brevis (APB) (thenar MUNE). This muscle predominately relates to thumb abduction, and therefore only partially contributes to grip strength it is a distal muscle that can be used to determine whether loss of functioning motor neurones is occurring distally. The thenar muscles are vulnerable to common disorders like carpal tunnel syndrome, which may result in handgrip weakness [17]. To exclude this, median motor nerve conduction studies were performed before each MUNE study. The presence of carpal tunnel syndrome was defined as a median distal motor latency of greater than 4.0 ms [17].

Self-adhesive surface recording electrodes (G1) were placed transversely across the innervation zone of the right APB, resulting in a simple biphasic negative-positive M wave, with G2 placed over the distal phalangeal joint. The median nerve was stimulated at the wrist with a surface stimulator. This was performed by strapping the stimulating electrode onto the surface of the skin, at the point where the threshold of the nerve to electrical stimulation was at its lowest. A hand-held stimulator was not used, as reproducibility is enhanced when the stimulating electrodes are fixed to the surface of the skin. Median nerve stimulation at the wrist was well tolerated by all subjects, as the stimulation intensity required to obtain an adequate response was generally less than 20 mA with duration of 0.1 ms.

Maximum isometric grip strength was obtained using the Jamar hydraulic dynamometer at the standardised, middle handle position with consistent instructions given to the 69 subjects. Handgrip force was measured with subjects sitting with their arm flexed at 90 degrees. The right hand was tested first and then the left. 3 trials were performed on each hand, and the highest result was recorded. This technique was used as studies have shown no significant difference in reliability between one attempt, mean score of two or three attempts, or the highest score of 3 attempts [12].

Clinical neurological examination was performed on all 69 subjects, with the strength of thumb abduction, finger flexion and finger abduction measured according to the Medical Research Council (MRC) grading system.

All statistical analysis was performed with SPSS 10.0 for Windows. The correlation between hand grip and MUNE was measured using the Pearson Product Moment Correlation, as the Pearson's coefficient reflects the degree of linear relationship between two variables.

## 3. Results

**Figure 1:**
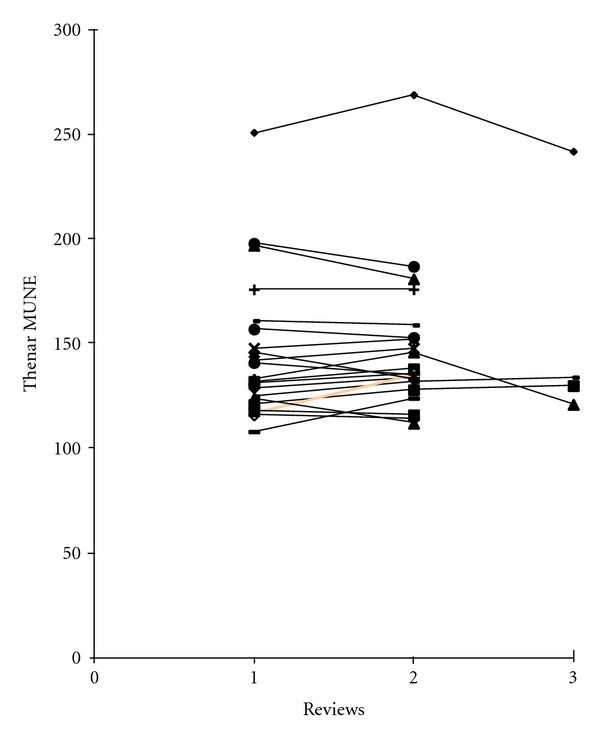
Reproducibility of thenar MUNE in population controls on separate reviews over a one-year period.

Isometric grip strength tests, thenar MUNE, and MRC power were performed on 69 asymptomatic subjects. The technique was found to be reproducible, in our previous study indicating that the test-retest correlation of thenar MUNE in these 69 asymptomatic subjects was high, with a Pearson correlation coefficient of 0.93. The mean difference between MUNE results on separate occasions on the same individual was ±3.6%, with a range of 0–11.7% [13] (Figure 1).

**Figure 2:**
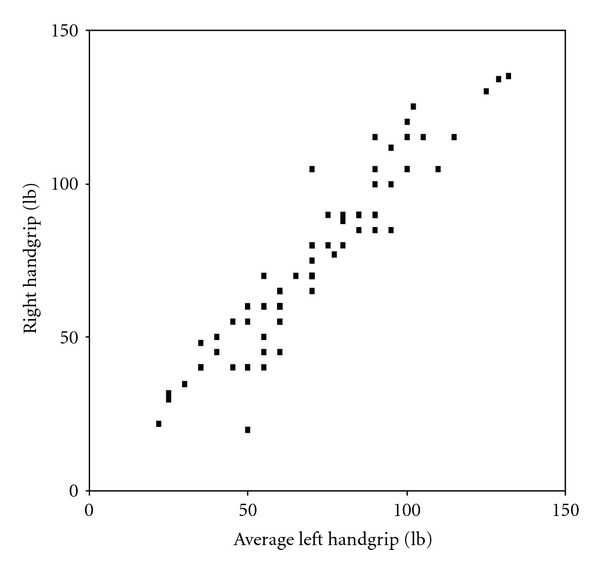
Scatter graph showing the correlation between right and left handgrip.

Right-hand grip strength correlated with left-hand grip strength, with Pearson's correlation coefficients of 0.945 (*P* < 0.001). The ranges for right- and left-grip strength were similar right 20–135 lb with a mean on 75.04 lb and left 22–132 lb with a mean of 71.14 lb. Two-way analyses of variance showed a no-significant difference between the right and left hands, Figure 2.

**Figure 3:**
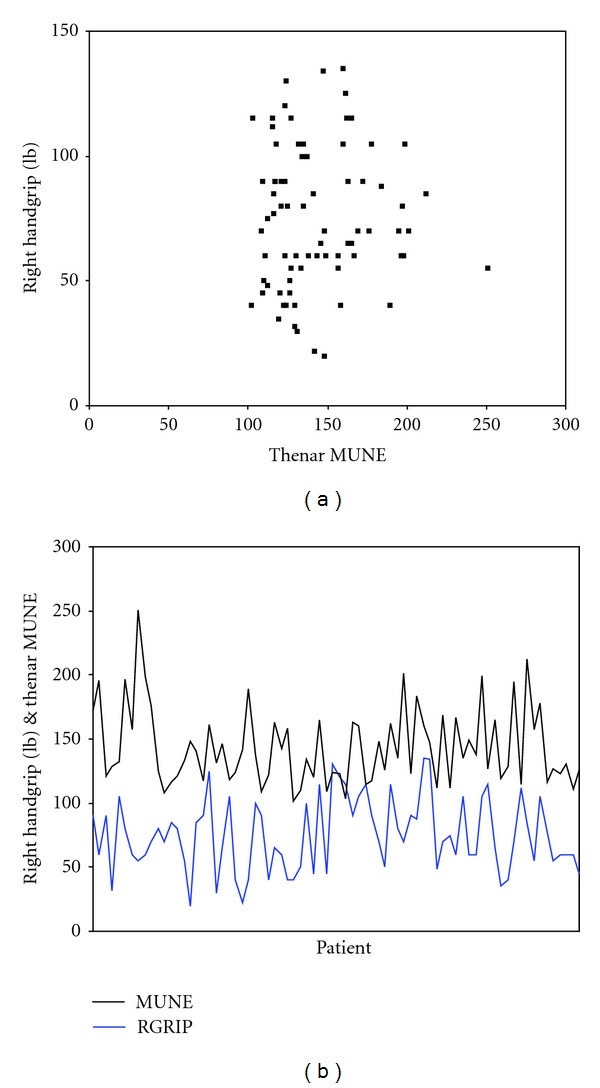
Scatter and line graph showing the lack of correlation between right handgrip and thenar (APB) MUNE.

Right maximum isometric grip strength using the Jamar hydraulic dynamometer correlated poorly with the number of functional motor neurones in thenar group of muscles as measured using the statistical method of MUNE, with a Pearson correlation coefficient of only 0.028 (Figure 3).

Power right thumb abduction, finger flexion, and finger abduction was measured according to the Medical Research Council (MRC) grading system and was found to be of grade 5/5 in all subjects.

## 4. Discussion

This study shows that there is a lack of correlation between handgrip MVIC and thenar MUNE, indicating that handgrip strength is not as sensitive as thenar MUNE for monitoring disease progression, as it is unable to detect early motor neurone loss due to the presence of compensatory mechanisms. The surviving motor neurones enlarge territories, through collateral sprouting (reinnervation) until late in the disease, when collateral reinnervation is no longer able to provide full functional compensation and weakness occurs [3]. MUNE may be reduced because of remodelling of the motor units, but in this study, CMAP amplitudes were maintained despite a reduction in MUNE. This may be because, early in the disease, the rate of cell death is low and not detectable [5].

This implies that MUNE may be a reliable and sensitive method for diagnosing MND. MUNE can be used as a noninvasive method of predicting impending decline in motor neurones and estimating the rate of neuronal death in asymptomatic subjects. Analysis of the density of the electromyographic interference pattern during maximal effort is not quantitative and requires the full cooperation of the patient.

MUNE has been performed in a number of different ways, each with their advantages and limitations [18]. The choice of technique depends largely on the speed and simplicity of the technique, as well as its accuracy and reproducibility. Each method measures the average SMUP and the size of the CMAP obtained with maximal stimulation of a motor nerve [19]. Most employ electrical stimulation of the motor nerve to determine the sizes of the SMUP, but a few techniques use needle EMG. The choice of technique largely depends on the speed and simplicity of the technique, as well as its accuracy and reproducibility.

The statistical MUNE technique used in this study has been compared to the multiple point stimulation method and found to be more reproducible (7% versus 12%) and faster to administer [20]. The technique has been greatly modified since its original description [15], but, despite this, numerous studies have shown that MUNE can be used to monitor change in MND patients when used by experienced technicians [14].

There have been studies performed indicating that the statistical MUNE is unreliable in the presence of clinical weakness due to motor unit instability and have suggested that the multipoint incremental method is more reliable as an outcome measure in ALS [21]. From our previous study, we found that the statistical method of MUNE method was fast, noninvasive, and reproducible, so that results obtained could be used as a baseline to follow loss of motor units over time, with estimates having coefficients of variability in the order of ±5% [13]. In addition, all subjects had normal Medical Research Council (MRC) grade 5/5 power in thumb abduction, finger flexion, and finger abduction, so the criticism of this technique was not a concern.

Generally, however, manual motor testing used in a standard neurological motor examination does not allow objective documentation of change in performance, as it may be influenced by the patient's history and progress. Major changes are apparent, but subtle changes are difficult to determine with accuracy. To improve this, a number of methods have been developed to quantify MVIC. The hand-held Jamar hydraulic dynamometer has been proposed as a clinically useful, reliable, reproducible, time efficient, and quantitative measure for monitoring disease progression in MND [10]. There have also been studies investigating the reliability of isometric grip strength testing, and more recently, normative maximum grip strength data for men and women has been developed to help clinicians interpret results obtained [12].

Thumb abduction power correlates better with thenar MUNE, but the Jamar dynamometer is not able to determine strength of isolated functional groups of muscles. Also, the thenar MUNE does not examine all of the motor units involved in handgrip MVIC, as forearm flexors and ulnar-innervated muscles are also involved. Finally, another explanation for the lack of correlation may be that, in MND, there is not uniform motor neurone loss in all parts of peripheral motor nerves.

In our previous study, we showed that MUNE was able to identify a deterioration in functional motor units before clinical weakness became apparent [22]. This confirms McComas' observation that patients with substantial chronic denervation could maintain normal muscle twitch tension until loss of about 50–80% of motor units occurs [3]. Felice showed that, in 21 MND patients, change in thenar MUNE was the most sensitive outcome measure for following disease progression, when compared to other quantitative tests, such as CMAP, isometric grip strength, forced vital capacity, and Medical Research Council manual muscle testing [23].

This study suggests that there is a lack of correlation between handgrip MVIC and thenar MUNE, implying that MUNE may be a reliable and sensitive method for diagnosing MND. MUNE can be used as a noninvasive method of predicting impending decline in motor neurones and estimating the rate of neuronal death in asymptomatic subjects.
